# Analyzing factors associated with time to age at first marriage among women in Ethiopia: log logistic-gamma shared frailty model

**DOI:** 10.1186/s12905-022-01775-1

**Published:** 2022-05-25

**Authors:** Molalign Gualu Gobena, Yihenew Mitiku Alemu

**Affiliations:** Department of Statistics, Natural and Computational Sciences, Assosa University, P. Box 18, Assosa, Ethiopia

**Keywords:** Clustering, Random effects, Heterogeneity, Laplace transformation, Frailty, Multilevel

## Abstract

**Objective:**

The main objective of this study is to fit Log logistic-Gamma shared frailty model for the determinant of time to age at first marriage among women in Ethiopia.

**Methods:**

The data set in this study were obtained from Demography and Health survey conducted in Ethiopia in 2016. In this study, we used Log logistic-Gamma shared frailty model to account for the loss of independence that arises from the clustering of women in region of Ethiopia. A total of 12,066 women aged 15–49 in Ethiopia were included in this study.

**Results:**

Of all 12,066 women aged 15–49, 9466 (78.45%) were married and the median & mean age at first marriage for women living in Ethiopia were 17.2 years and 17.5 years respectively, while the minimum and maximum age at first marriage observed were 8 years and 49 years respectively.

**Conclusion:**

The most significant contributing factors to delaying time to age at first marriage of women aged 15–49 in Ethiopia were increased education level of women, increased education level of the head, increased income, residing in urban and being followers of religion other than orthodox, catholic, protestant & Muslim. The heterogeneity of age at first marriage for women aged 15–49 among regions in Ethiopia was observed. The government of Ethiopia and the concerned bodies should revise the women's health policy and practice to reduce early marriage and give attention to women; illiterate, live in rural areas, and have illiterate and poor heads.

## Introduction

Time to age at first marriage is length of time from birth until the age by which the individuals have been married for the first time [1, 2]. For individuals, it is the length of time up to a new chapter of life [1, 3]. The couples may lead a new chapter of life by strengthening their economy, and they may have children over time thru birth or adoption [2, 3]. In Ethiopia, women tend to have tremendously less time to age at first marriage than men. The median age at first marriage is 16.5 years among women age 25–49 and 23.1 years among men age 25–49. 58% of women and only 9% of men age 25–49 marry before their 18th birthday [4, 5]. Our main motivational factors to conduct a depth study on time to age at first marriage is that the effects that they could have on reproductive health and socio-economic pertaining issues in Ethiopia. This is due to the fact that, time to age at first marriage, especially among girls is often associated with adolescent motherhood, school dropouts, maternal morbidity and mortality, and forfeited future life opportunities for the affected individual[1, 6]. In Ethiopia, a finding from study in Injibara town reveals that, 44.8% of women in Injibara town had first married below 18 years. In this area, the mean age at first marriage was 17 years, while the minimum and maximum age at first marriage was 9 and 23 years respectively [7]. The result from another study in Ethiopia particularly, in Dabat district shows that 86.6% of women in Dabat district had first married below 18 years. These studies concluded that women in these areas had first married at earlier time [8].

Most study in Ethiopia conducted using logistic regression and Cox proportional hazard models to analysis the determinant of time to age at first marriage [4, 5]. But, those models have their own amiss and cannot be applied for multilevel survival data. For instance, logistic regression does not provide methods for survival data and a Cox proportional hazard model require independency assumption of samples [9, 10]. To overcome this situation, we used Log logistic-Gamma shared frailty model. This model permits the analysts to account for the loss of independence that arises from the clustering of subjects in higher level units. Similarly, it allows researchers to make valid inferences when examining the effect of both subject characteristics and cluster characteristics on the risk of the occurrence of the outcome. It is a model that incorporates cluster-specific random effects to account for within cluster homogeneity in outcomes [9]. For instance, in this study women with in the same region share the same random effect with respect to marriage. As a result, here we used a Log logistic-Gamma shared frailty model for our two level data.

The general objective of this study is to analysis factors associated with time to age at first marriage among women in Ethiopia using advanced methodology. Distinguishing significant associated factors with time to age at first marriage among women in Ethiopia and estimating the clustering effect in modeling time to age at first marriage for the data set are the specific objectives of this study. Conducting this study has a great role in designing women's health policy and identifying priority health programs in Ethiopia. The study also set a solution for women’s health-related problems in Ethiopia.

## Methods

### Study design and setting

This study used a population-based cross-sectional design and data from 2016 Ethiopian Demographic and Health Survey (EDHS), which was gathered between January 18 and June 27, 2016. The purpose of the survey was to give estimates for demographic and health predictors of interest in Ethiopia's two administrative cities and nine geographical regions. The survey included 12,066 women between the ages of 15 and 49.

### Variable in the study

Time to age at first marriage (dependent variable) is a measured value in years from birth to age at first marriage. On a sample of all women aged 15–49 in Ethiopia, we retrospectively observe the timing to first marriage since birth. Hence we have to consider two things. First, all cases with no observed events are right censored. Therefore the women who had not yet experienced the event of interest resulting in right censoring of the data. Second, in order to make censoring valid, we have to assume that all women marry before the age of 50.

Wealth index, media exposure, head/parents' occupations, women's education level, head/parents' education level, type of residence, religion, and Respondents' work status are the independent variables investigated in this study. In the frailty model we have considered regional state of the women as a clustering effect.

## Method of data analysis

### Multilevel survival analysis

Multilevel survival analysis is the statistical technique that can apply for clustered (grouped) survival times. Researchers often encounter grouped or multilevel data like individuals are nested within families, and families are nested within neighborhoods. In our study also encountered such kinds of data. For instance women aged 15–49 nested with in region i.e. we have a two level data. Analyzing such data requires special treatment because most multivariate models assume that observations are independent, and grouped data clearly violate this assumption. Statisticians and biomedical researchers identified adverse consequences of applying the Cox regression to grouped survival times [11, 12]. They noted that when the independent assumption of the Cox model is violated, the tests of statistical significance are biased and in ways that cannot be predicted beforehand [13]. Since Log logistic-Gamma shared frailty model is one of the statistical models for multilevel survival analysis [10], we used it in this study.

### Modeling frailty and shared frailty model

A model for multilevel survival data may consider the heterogeneity among individual failure times and the heterogeneity between groups of failure times. If we add a random effect from the proportional hazards model the resulting model is called the frailty model. The inclusion of this random effect in this model helps to measure the heterogeneity among individuals. The term “frailty” is used to express heterogeneity. In addition, to explicitly measure the heterogeneity among individuals, this model allows measuring truly the effects of covariates on the hazard function of individuals [14, 15]. In another way, if we add a group-specific random effect from the proportional hazards model the resulting model is called the shared frailty model. This model is used to know the heterogeneity among individual failure times and the heterogeneity between groups of failure times. This group-specific random effect can be considered as an unobserved covariate which is in common for that specific group. The term “shared frailty” comes from the concept that individuals within the same group share the same heterogeneity [9, 14, 15].

If G and n_i_ represents the number of groups and individuals in ith group respectively, the hazard function of the jth individual in ith group is.

1 $$h_{ij} (t/ \, Z_{ij} ) = \, h_{0} \left( t \right)\exp \left( {\beta Z_{ij} + \psi W_{i} } \right), \, i = 1, \ldots .G,j = 1, \ldots , \, n_{i}$$

where w_i_ is ith group unobservable covariates, ψ is regression coefficient for ith group unobservable covariates, Z_ij_ is the jth individual observable covariate vector in ith group. exp(ψw_i_) is the frailty of the ith group and if we replace it by u_i_, the hazard function can be simplified as:

2 $$h_{ij} (t/ \, Z_{ij} ) = \, h_{0} \left( t \right) \, u_{i} \exp \left( {\beta Z_{ij} } \right), \, i = 1, \ldots .G,j = 1, \ldots , \, n_{i}$$

The random variables u_1_, . . . ,u_G_ assumed to have a common probability density function q.

This random effect model uses u as a random effect to represent group variation and the hazard function h_0_(t) exp(βZ_ij_) to represent individual variation. When a group's frailty value is high, the group experiences the event of interest at earlier times [9, 14, 15].

### The gamma frailty model

In gamma frailty model, the frailty assumed to have a gamma distribution. It fits failure time very well. Mathematical tractability and analytical simplicity are the two main benefits of this model. Those benefits are the result of mathematical derivative-able of the Laplace transformation of the model [15, 16].

A frailty's density function in one parameter gamma distribution (θ) is.

3 $${\text{q}}\left( {\text{u}} \right) \, = \, [{\text{u1}}/\theta - {\text{1 exp}}( - {\text{u}}/\theta )\left] / \right[\Gamma ({1}/\theta )\theta {1}/\theta ]$$

If θ > 0 and u > 0, individuals in group experience the event of interest at earlier times (more frail). If u < 0, individuals in group experience the event of interest at latter times (less frail).The Kendall's Tau [9] measure the relationship between any two events times within group for the Gamma distribution and is given by:-

4 $$\tau \, = \theta / \, (\theta + {2}),\;{\text{where}}\;\tau \in \left( {0,{1}} \right)$$

The Laplace transform of the model in (2.3) is expressed as; *LP(s)* = *(1*+θs)^−1/θ^.

### Log logistic-gamma shared frailty model

In Log logistic-Gamma shared frailty model, the baseline hazard assumed to have a log-logistic distribution and the shared frailty assumed to have a gamma distribution [17–19].

### Log-logistic distribution for Baseline hazard

The log-logistic distribution is a hybrid of the Gompertz and Gamma distributions, with the mean and variance values equal to one [20, 21].Various functional forms for f(t), S(t), h(t) & H(t) in log-logistic distribution are presented in (Table 1).

### Results

#### Descriptive statistics

From a total of 12,066 women aged 15–49 in Ethiopia, 9466(78.45%) were married. The median & mean age at first marriage among women aged 15–49 were 17.2 years and 17.5 years respectively. In addition, the minimum and maximum age at first marriage observed were 8 years and 49 years respectively. The highest and lowest percentage of women aged 15–49 who were first married were in the Amhara region (11.14%) and in the Somali region (4.81%) respectively. Both median and mean age at first marriage among women in Addis Ababa was 19 years which was the highest relative to other regions. In contrast, the median and mean age at first marriage among women in the Amhara region was 14 years and 14.5 years respectively which were the lowest relative to other regions. Women aged 15–49 who were first married and reside in the rural part of Ethiopia were higher in percentage (57.97%) than those residing in the urban area (20.48%). Both median and mean age at first marriage among women in the urban part of Ethiopia were 18 years and 18.5 years respectively which were higher than women in the rural area (median = mean = 16 years).

Regards to the educational level of women, the percentage of illiterate women aged 15–49 who were first married was highest (50.39%), while the lowest for women at higher education level (2.54%). The median and mean age at first marriage among women aged 15–49 are 23 years and 22 years respectively for those women who achieved higher education, which is highest, while lowest for illiterate women (mean = 15, median = 17). The finding also shows that women aged 15–49 who were first married and followers of the Muslim religion in Ethiopia were highest in percentage (31.87%), while it is lowest for catholic religion followers (0.84%).

Both the mean and median age at first marriage among women aged 15–49 in Ethiopia was 18 years which is similar and highest for women who follow a religion other than orthodox, Muslim, catholic, and protestant, while it is lowest for the orthodox follower (mean = 16, median = 17).

The percentage of women aged 15–49 who were first married was highest for the women that having richest head (21.95%) and followed by the women that having poorest head (20.45%), while the lowest for those having a middle income head (11.78%). Median and mean age at first marriage were highest and similar for 15–49 aged women who having richest head in Ethiopia, while the lowest for those having poorest head (mean = 16.5, median = 17). The result also shows that the percentage of women aged 15–49 who were first married was highest for those women having an illiterate head (39.87%) and agriculturalist head(50.91%), while lowest for those having a better-educated head (4.91%) and professional head (4.46%). However, median age and mean age at first marriage are similar as well as highest (20 years) for those women having head who achieved higher education, while similar as well as lowest for those of having no educated head (16 years).

The percentage of women aged 15–49 who were first married was higher for media-accessible women (48.22%) than those who haven’t (30.23%). Median age and mean age at first marriage for women aged 15–49 are higher among those having better access to media and wealthier. The median age at first marriage among women aged 15–49 in Ethiopia was 18 years for women having professional and laborers head which were the highest, while the lowest for women having an agricultural head (16 years). Similarly, the mean age at first marriage was highest for women having a professional head (18.5%) and lowest for women having an agricultural head (16%).

Finally, the percentage of women aged 15–49 who were first married was higher for those who haven't a job (work) (50.53%) than those having work (27.92%). The median and mean age at first marriage is similar, which is 16 years irrespective of work status (Table 2).

**Table 1:** Functional forms for f(t),S(t), h(t) & H(t) in log-logistic distribution [17–19]

Baseline distribution	f(t)	S(t)	h(t)	H(t)	Parameter space
Log-logistic	(λγt^γ−1^)/(1 + λt^γ^)^2^	1/(1 + λt^γ^)	(λγt^γ−1^)/(1 + λt^γ^)	Ln[1 + (t/λ)^γ^]	λϵR, γ > 0

f(t) = probability density at a time t, S(t) = Survival at a time t, h(t) = hazard at a time t, H(t) = cumulative hazard at a time t

### Analysis based on log logistic-gamma shared frailty model

Analysis based on this model showed that place of residence of women, religion of women, education level of women, some categories at wealth index of household and head education were significant at 5% level of significance (*P *value < 0.05). In contrast work status of women, head occupation and access to media were not significant at 5% level of significance (*P *value  > 0.05).

An odds ratio of greater than 1 indicates delaying the time to age at first marriage. Therefore women who attend primary school (ϕ = 1.034), secondary school (ϕ = 1.183) and higher education (ϕ = 1.325) had delayed age at first marriage by a factor of 1.034, 1.183 & 1.325 respectively than the reference category (No Education). Concerning religion of women, women who are follower of catholic; protestant, Muslim and other religion (religion except orthodox, catholic, protestant & Muslim) had delayed age at first marriage by a factor of 1.047, 1.05389, 1.05387 and 1.067 respectively when compared to the reference category (orthodox). Head education is also another significant covariate with odds ratio greater than 1 for all categories. As a result those women having head that attend primary school, secondary school and higher education had delayed age at first marriage by a factor of 1.016, 1.029 and 1.044 respectively when compared with the reference category (No Education). Categories of Significant covariates having odds ratio less than 1 imply that women characterized by those categories of the same covariate had less delayed age at first marriage relative to the reference category of the same covariates. For instance, women residing in rural area of Ethiopia have less expected survival time (duration of age at first marriage) than those residing in urban area (ref) of Ethiopia (ϕ = 0.967). Similarly, richer (ϕ = 0.970) and richest women (ϕ = 0.949) had less delayed age at first marriage than reference category (poorest women). Here, we see that poorer and middle categories are not statistically significant at 5% level of significance (*P *value  > 0.05).

The value of the shape parameter in the Log logistic –Gamma shared frailty model was (γ = 0.131). This indicates that non monotonic hazard rates, specifically initially increasing and then decreasing rates. The variability (heterogeneity) of age at first marriage in women population of clusters (Region) estimated by this Log logistic –Gamma shared frailty model was* θ* = 0.088 and the dependence within region were about τ = 4.2% (Table 3).

**Table 2:** Descriptive summary for women age at first marriage by categories of covariates, EDHS 2016

Covariates	Category	No. (percentage) of Women	No.(percentage) of women who were first married	Mean (Year)	Median (Year)
Region	Tigray	1281 (10.62)	959 (7.95)	16.5	16
	Afar	1084 (8.98)	885 (7.33)	16.5	17
	Amhara	1630 (13.51)	1344 (11.14)	14.5	14
	Oromiya	1576 (13.06)	1140 (9.45)	16.5	17
	Somali	741 (6.14)	580 (4.81)	17	17
	Benshangul- Gumeze	1025 (8.49)	867 (7.19)	16	16
	SNNP	1436 (11.9)	1042 (8.64)	18	18
	Gambela	927 (7.68)	742 (6.15)	17	16
	Harari	768 (6.36)	626 (5.19)	17	17
	Addis Ababa	846 (7.01)	654 (5.42)	19	19
	Dire Dewa	752 (6.23)	627 (5.20)	18.5	18
Residence	Urban	3145 (26.06)	2471 (20.48)	18.5	18
	Rural	8921 (73.94)	6995 (57.97)	16	16
Education level	No Education	7647 (63.38)	6080 (50.39)	15	17
	Primary	3331 (27.61)	2580 (21.38)	17	18
	Secondary	657 (5.45)	499 (4.14)	19	21
	Higher	431 (3.57)	307 (2.54)	22	23
Religion	Orthodox	4765 (39.49)	3714 (30.78)	16	17
	Catholic	132 (1.09)	101 (0.84)	17	18
	protestant	2129 (17.64)	1642 (13.61)	17	18
	Muslim	4838 (40.10)	3846 (31.87)	17	18
	Others	202 (1.67)	163 (1.35)	18	18
Wealth Index	Poorest	3116 (25.82)	2468 (20.45)	16.5	17
	Poorer	1935 (16.04)	1491 (12.36)	16.5	17
	Middle	1798 (14.90)	1421 (11.78)	16.5	17
	Richer	1848 (15.32)	1438 (11.92)	16.5	17
	Richest	3369 (27.92)	2648 (21.95)	18	18
Head education	No Education	6040 (50.06)	4811 (39.87)	16	16
	Primary	4080 (33.81)	3172 (26.29)	16.5	18
	Secondary	1152 (9.55)	891 (7.38)	18	19
	Higher	794 (6.58)	592 (4.91)	20	20
Work status	No	7734 (64.10)	6097 (50.53)	16.5	17
	Yes	4332 (35.90)	3369 (27.92)	16.5	17
Access to media	No No	7300 (60.50)	5818 (48.22)	16.5	16
	Yes Yes	4766 (39.50)	3648 (30.23)	17	17
Head occupation	Agriculturalist	7822 (64.83)	6143 (50.91)	16	16
	Professional	706 (5.85)	538 (4.46)	18.5	18
	Laborers	1193 (9.89)	931 (7.72)	17.5	18
	Business	1384 (11.47)	1099 (9.11)	17.5	17
	Others	961 (7.96)	755 (6.26)	17.5	17

**Table 3:** Multivariable analysis using the Log logistic-Gamma shared frailty model, EDHS 2016

Covariate	Coeff	St.err	*P*-value	*ϕ*
Constant	2.757132	0.014421	** ≤ 0.001**	15.75
**Residence**				
Urban(ref)				
Rural	− 0.0331585	0.0097971	** ≤ 0.001**	0.9673852
**Education level**				
No education(ref)	1			
Primary	0.0330889	0.005878	** ≤ 0.001**	1.0336424
Secondary	0.1684082	0.0131558	** ≤ 0.001**	1.1834196
Higher	0.281212	0.0163527	** ≤ 0.001**	1.3247344
**Religion**				
Orthodox(ref)				
Catholic	0.046315	0.0227856	**0.042**	1.0474043
Protestant	0.0524916	0.0081184	** ≤ 0.001**	1.0538937
Muslim	0.0524736	0.0064776	** ≤ 0.001**	1.0538747
Others	0.0647476	0.0185256	** ≤ 0.001**	1.0668897
**Wealth index**				
Poorest(ref)				
Poorer	− 0.0099705	0.0072415	0.169	0.9900790
Middle	− 0.014243	0.0074423	0.056	0.9858580
Richer	− 0.0299688	0.0075612	** ≤ 0.001**	0.9704758
Richest	− 0.0528073	0.0107207	** ≤ 0.001**	0.9485628
**Head education**				
No education(ref)				
Primary	0.0154898	0.0055625	**0.005**	1.0156104
Secondary	0.02867	0.010067	**0.004**	1.0290849
Higher	0.04283	0.0140958	**0.002**	1.0437604
**Work status**				
No(ref)				
Yes	− 0.0046146	0.0049996	0.356	0.9953960
**Access to media**				
No(ref)				
Yes	− 0.0003079	0.0057177	0.957	0.9996921
**Head occupation**				
Agriculturalist(ref)				
Professional	− 0.0078202	0.0126729	0.537	0.9922103
Laborers	0.0195623	0.0099795	0.050	1.0197549
Business	0.0117877	0.0085707	0.169	1.0118574
Others	− 0.0065789	0.009861	0.505	0.9934427
Random effects				
γ = 0.131, *θ* = 0.088, τ = 4.2%				

*P *value  = *P *value for Likelihood-ratio test of theta = θ at chi-square with 0 and 1 degrees of freedom, θ = theta (variance of random terms), τ = Kendaell’s tau, Coeff = Coefficient of factors, St.err = Standard error of coefficient, ϕ = Odds Ratio

Bold values indicate to make the identification of included covariates in the study simple for readers and to easily distinguish the significant covariates

## Discussion

At 5% level of significance, the results from Log logistic-Gamma shared frailty model shows that, residence of women, educational level of women, religion of women, wealth index of household and head educational level are the most significance factors in predicting time to age at first marriage, whereas work status of women, access to mass media, and head occupation are not. A study by Haloi [22] revealed that wealth index of household and head education is significant factors in affecting time to age at first marriage. This result coincides with our findings. Moreover, the result from our study shows that, women who had better educated parent have delayed age at first marriage than women who had least educated parent. With respect to wealth index, women who had richest parent have less delayed age at first marriage than women who had poorest parent. This might be explained from the fact that, the increment of education level and income of household head leads to the increment of the perception and knowhow of household head for the consequence of early marriage. In addition, in Ethiopian context, household head have a direct or indirect involvement in deciding the age of first marriage for their daughters. This implies wealthier and educator household head have a great role in delaying age at first marriage.

The finding from a study by Adebowale [23] revealed that residence of women, educational level of women and religion of women had a significant effect on age at first marriage. This result also coincides with our findings. In our finding, better educated women had delayed age at first marriage than least educated women. The possible reason might be the crucial role of education in creating and developing knowledge and attitude towards the impact of early marriage. Concerning to religion of women, orthodox follower women had less delayed age at first marriage than other religion follower’s women. This finding is in line with the fact that, preachers in orthodox religion of Ethiopia didn’t focused on religion education about marriage. So that, their followers might be in lack of awareness about the impact of early marriage and then have less delayed age at first marriage. With regards to residence of women, women residing in rural area of Ethiopia have less expected time to age at first marriage than those residing in urban area of Ethiopia. In Ethiopian context, parents in rural area take early marriage as a cultural set up to avoid sexual risk (unintended pregnancy, rape and abduction) of their children. In addition, women in rural area are less educated and have no decision power for their age at first marriage. According to Von [24] findings work status of women, educational level of women and residence of women had a significant effect on age at first marriage but not head occupation of women. This finding contradicts our finding with respect to work status. Access to mass media was found to have a significant effect on age at first marriage by Zahangir [25]. But, in our study it was not. The findings from another study in Ethiopia reveals that, place of residence of women, head occupation, education level of women, work status of women, and head education have a significant effects on time to age at first marriage, while religion of women, access to media of women, and wealth index of a household were not have a significant effect [26]. This seems a similar study with differed statistical analysis from the present study. But, the parameter estimate in the present study is more precise which leads to more accurate statistical inference than its counterpart. This is because the present study assumes a parametric distribution for the baseline hazard function of the model, while this previous study doesn’t assume. In another way, the findings from the present study are more useful for making effective decision relative to this previous study.

## Strengths and limitations of this study

- The study provides information on marriage in Ethiopian women by analyzing the impact of different covariates on time to age at first marriage using advanced methodology.
- The study put a solution for women’s health-related problems in Ethiopia and gives input for designing women health-related policy and practice in Ethiopia.
- The study verifies whether there is heterogeneity in time to age at first marriage for women in Ethiopia among regions in Ethiopia.
- All low and middle-income countries may not have the same cultural norms, traditional beliefs, and moral rules for the early marriage of girls. In addition, they may not have a similar socio-economic condition. As a result, our study findings may not be conclude-able for those countries.
- There may be recall bias when respondents respond to the questionnaire about time-to-age at first marriage.

## Conclusion

In this study, we were considered various characteristics of women to analyze their effects on time-to-age at first marriage among women aged 15–49 in Ethiopia by using the Log logistic-Gamma shared frailty model. However, the most significant contributing factors to delaying time to age at first marriage of women aged 15–49 in Ethiopia were increased education level of women, increased education level of the head, increased income, residing in urban and being followers of religion other than orthodox, catholic, protestant & Muslim. In this study, the work status of women, access to mass media, and head occupation were not having a significant effect on time to age at first marriage. The model reveals the presence of a significant cluster (frailty) effect. This implies the heterogeneity of age at first marriage for women aged 15–49 among regions in Ethiopia. The government of Ethiopia and the concerned bodies should revise the women's health policy and practice to reduce early marriage and give attention to women; illiterate, live in rural areas, and have illiterate and poor heads.

## Data Availability

After a brief registration and explanation of the data's intended use from the online form of Ethiopian CSA, the data freely available at http://dhsprogram.com/data/dataset/Ethiopia_Standard-DHS_2016.cfm?flag=0

## Abbreviations

CSA: Central statistical agency
DHS: Demographic and health survey
EDHS: Ethiopian demographic and health survey
